# Abstracts

**DOI:** 10.1155/S1463924686000214

**Published:** 1986


Journal of Automatic Chemistry/*Journal of Clinical Laboratory Automation*, Vol. 8, No. 3 (July–September 1986), pp. 101–105

### Abstracts

### Page 106

### Automated chemical synthesis. Part 4: Batch-type reactor automation and real-time software design

D. F. Chodosh *et al.*

Rapid enhancements in computer technology promise computing instrumentation with greater speed and capacity at ever more attractive price/performance. These engineering advances challenge our ability to usefully apply these extraordinary new tools in the research laboratory. In the authors' research program, focusing a special emphasis on the interaction of the laboratory scientist with computing systems, a bench-scale batch-type chemical reactor capable of self-directed experimentation has been developed. Through interactive dialogue, the chemist establishes stochiometry, temperature and reaction endpoint criteria; the automated synthesis system then executes the requisite experiments: delivering starting materials and solvents to the reactor, maintaining temperature setpoints, sampling reaction aliquots as required for online HPLC analysis. As the chemical analysis results are available to the system in real-time, the instrument is capable of self-directing experimentation and can employ experimental design strategies including Simplex optimization and factorial design schema.

### La synthèse chimique automatisée, partie 4: Automatisation d'un réacteur 'batch' et conception d'un logiciel en temps réel

D. F. Chodosh *et al.*

Les grands progrès en technologie d'ordinateur promet des instruments avec plus grande capacité et vitesse à prix de plus en plus intéressant. Ces progrès technologiques sont une motivation pour appliquer ces nouveaux instruments extraordinaires dans le laboratoire de recherches. Dans notre programme de recherches, l'interaction du scientifique de laboratoire avec les systèmes d'ordinateur nous semble spécialement important, nous avons développé un réacteur chimique pour laboratoire capable d'effectuer des expériences automatiques. Utilisant un dialogue interactif le chimiste établit la stoechiométrie, la tempé rature et les critères de fin de réaction; le système de synthèse automatique réalise les expériences requises: c'est-à-dire addition des matériaux de départ et des solvants dans le réacteur, maintient la température requise, et échantillonne les parties aliquotes requises pour l'analyse HPLC en ligne. Du fait que les résultats d'analyse chimique sont accessible au système en temps réel, l'instrument est capable de contrôler soi-même les paramètres de l'expérience et peut même utiliser des stratégies de conception expérimentale du genre optimisation Simplex et schéma factoriel.

### Automatisierte chemische Synthese. Teil 4: Batch-Reaktor Automation und Echtzeit-Software Aufbau

D. F. Chodosh *et al.*

Die schnellen Entwicklungen in der Computertechnologie versprechen Computerinstrumentierung mit höherer Geschwindingkeit und Kapazität bei immer attraktiverem Preis-Leistungsverhältnis. Diese technischen Fortschritte fordern uns heraus diese neuen ausserordentlichen Werkzeuge im Forschungslabor nützlich anzuwenden. In unserem Forschungsprogramm, in dem wir der Interaktion des Laborwissenschaftler mit Rechensystemen spezielle Aufmerksamkeit widmen, haben wir einen chemischen Labor-Batchreaktor entwickelt, der selbstgesteuerte Versuche durchführen kann. In einem Dialog legt der Chemiker die Stöchiometrie, Temperatur und die Reaktionsendpunkt-Kriterien fest; das automatische Synthesesystem führt daraufhin die erforderlichen Experimente durch: Dosieren der Ausgangsmaterialien und Lösungsmittel in den Reaktor, Steuerung der Temperatur, Entnahme von aliquoten Teilen des Reaktionsgutes zur on-line HPLC-Analyse. Sowie die Resultate der chemischen Analyse in Echtzeit für das System verfügbar werden, kann es die Selbststeuerung der Versuche übernehmen und experimentelle Versuchsstrategien wie z.B. die Simplex=Optimierung implementieren.

### Page 122

### Computer-aided chemistry. Part 1: Control of the PAR 273 electrochemical instrument using the IBM 9001 laboratory computer

T. Nyokong and M. J. Stillman

An extensive Fortran 77 computer program, ELECTRA, is described which acquires and displays data in real-time from the PAR 273 electrochemical instrument. The use of the IBM S9001 laboratory computer, which is based on the Motorola 68000 microcomputer chip, the IEEE 488 parallel port for fast data transfer, large amounts of random access memory and the high-resolution CRT display, means that the program provides an environment for the scientific user that, in essence, transfers the front panel control of the instrument to the computer's CRT display. Thus the program provides the user with complete interactive control of the electrochemical instrument within the operational limits of the equipment, using software-programmable keys and on-screen menus to prompt the user during the entry of set up and control information. Multiple graphics and display windows are used to display the running parameters, file names and data as it is acquired. The electrochemical experiments supported are cyclic voltammetry, differential pulse voltammetry, controlled potential coulometry and controlled potential electrochemistry.

**Computer-aided chemistry. Première partie: Contrôle de l'instrument électrochimique PAR 273 utilisant l'ordinateur de laboratoire IBM 9001**
T. Nyokong and M. J. Stillman

Un programme d'ordinateur en Fortran 77, ELECTRA, qui permet l'acquisition et la présentation de données en temps réel de l'instrument électrochimique PAR 273 est décrit. L'utilisation de l'ordinateur de laboratorie IBM S9001 qui se base sur le microordinateur Motorola 68000, la sortie parallèle IEEE 488 pour transfert de données rapide, un grand nombre de mémoires écriture/lecture RAM, un écran CRT à haute résolution signifie que, pour l'utilisateur scientifique, tous les contrôles man els de l'instrument ont été transférés à l'ordinateur. De cette façon, le programme permet à l'utilisateur le contrôle complet interactif de l'instrument électrochimique dans le domaine des limites opérationnelles de l'équipement, utilisant des touches programmables et la technique de menue pour guider l'utilisateur pendant l'introduction de paramètres. Différentes 'fenêtres' sont utilisées pour la présentation graphique ou numérique des paramètres actifs, des noms de fichiers et des données pendant leur acquisition. Les expériences électrochimiques utiliseés sont la voltamétrie cyclique, la voltamétrie à impulsion différentielle, la coulométrie à potentiel contrôlé et l'électrochimie à potentiel contrôlé.

**Computergestützte Chemie Teil I: Steuerung des PAR 273 Elektrochemie-Gerätes mit einem IBM 9001 Laborrechner**
T. Nyokong and M. J. Stillman

Es wird ein umfangreiches FORTRAN 77 Rechnerprogramm, ELECTRA, beschrieben, welches in Echtzeit Daten des PAR 273 Elektrochemie-Gerätes erfasst und darstellt. Die Benützung des IBM S9001 Laborrechners, welcher auf dem Motorola 68000 Mikroprozessor basiert, die IEEE 488 Parallelschnittstelle für raschen Datentransfer, ein grosser RAM Speicher und ein hochauflösender Bildschirm bedeuten, dass das Programm eine Benützerumgebung schafft, welches im wesentlichen die Steuerung von der Frontplatte des Gerätes zu Bildschirm und Tastatur verlegt. Somit ermöglicht das Programm dem Benützer mit Hilfe von programmierbaren Tasten und Bildschirmmenus, welche ihn während der Eingabe der Konfigurations- und Steuerinformationen führen, eine vollständige Steuerung des elektrochemischen Gerätes innerhalb der Betriebsgrenzen.

Vielfache Bildschirmfenster mit Grafik werden benützt, um die laufenden Parameter, Filenamen und Daten, wie sie erfasst werden, darzustellen. Die elektrochemischen Versuche, die gestützt werden, sind zyklische Voltametrie, Differential Plus-Voltametrie, gesteuerte Potentialcoulometrie und gesteuerte Potentialelektrochemie.

### Page 135

**Pipette cleaning in automated systems**
M. L. Severns *et al.*

Some of the factors which influence the cleaning of stainless-steel pipettes in automated sampling systems are investigated. In this study, the pipette was cleaned by inserting it into a 'wash station' and forcing distilled water through the bore of the pipette. The washout of contaminants from the pipette was found to be a second-order process which was strongly influenced by the volume of sample aspirated, and influenced to a lesser extent by the rate of flow of distilled water and the diffusibility of the analyte. Other factors which influenced the cleaning process were the position of the tip of the pipette in the wash station and the depth of the wash station. It is possible to clean a pipette adequately to perform assays whose dynamic range exceeds $10^6$.

**Le nettoyage de pipettes dans des systèmes automatisés**

M. L. Severns *et al.*

Quelques-uns des facteurs influençant le nettoyage des pipettes en acier inox pour les systèmes d'échantillonnage automatique sont étudiés. Dans cette étude la pipette est nettoyée en l'introduisant dans une 'station de nettoyage' et en forçant de l'eau distillée à travers la pipette. Le rinçage des contaminants de la pipette ressemble à un processus de second ordre fortement influencé par le volume d'echantillon aspiré et peu influencé par le débit de l'eau distillée et par la diffusibilité de l'échantillon. D'autres facteurs qui influencent le processus de nettoyage sont la position de la pointe de la pipette et sa profondeur dans la station de nettoyage. Il est possible de nettoyer les pipettes convenablement pour effecteur des dosages dans une plage dynamique excédant $10^6$.

**Reinigen von Pipetten in automatisierten Systemen**
M. L. Severns *et al.*

Es werden einige der Faktoren untersucht, die das Reinigen von Pipetten aus rostfreiem Stahl in automatisierten Probennahmesystemen beeinflussen. In der vorliegenden Studie werden die Pipetten durch Einführen in eine Waschstation und forciertem Durchspülen mit destilliertem Wasser gereinigt. Dabei stellte sich heraus, dass das Wegwaschen der Verunreinigung von der Pipette ein Prozess zweiter Ordnung ist, der stark durch das Volumen der angesogenen Probe und weniger durch die Durchflussgeschwindigkeit des destillierten Wassers und der Diffusionsfähigkeit des Analyten beeinflusst ist. Andere Faktoren, die den Reinigungsprozess beeinflussen, sind die Position der Pipettenspitze in der Waschstatin und die Tiefe der Waschstation. Es ist möglich, Pipetten zufriedenstellend zu reinigen, um damit Analysen mit einem dynamischen Bereich von mehr als $10^6$ durchzuführen.

### Page 142

**Automation in urinalysis: sample and data management, and quality control**
G. Barbaresi *et al.*

A procedure for daily management and quality control of urinalysis is described. Urine samples are analysed randomly on delivery to the authors' laboratory. Using the same random sequence, patient identification numbers are then entered in the minicomputer on line to the analysers. At the end of the analytical work it is possible to verify the reliability of analytical data and to sort the samples with abnormal qualitative results for glucose and protein. The results of microscopical examination of urine sediment are entered manually. A complete report of microscopical and analytical results is finally transferred to the Hospital Data Processing Center (HDPC) by means of a floppy disk. This HDPC, using identification numbers, links analytical results to patient data (name, surname etc.), and then prints out a complete, personalized report.

The reliability of analytical results is certified by an intra-laboratory quality control program based on the evaluation of results of control urine samples and on the analysis of patient results distribution.

*French and German abstracts of this paper will be published in our next issue.*

### Page 147

### A procedure for estimating bias between quantitative analytical methods
W. C. Griffiths *et al.*

If a new clinical laboratory analytical method is sufficiently precise and stable over a period of weeks, and is linear and free of carry-over, then a few simple operations can assess and document its degree of inaccuracy. That most methods are to some degree inaccurate does not invalidate their use. However, the burden is on the laboratory choosing a method to know how accurate the method is.

One operation can determine the presence and extent of bias between the method under study and a second method, the characteristics of which are presumably already known. An ample number of patient samples are analysed by both methods, preferably each of a pair being analysed on the same day. A plot of one method result versus the other is usually quite informative and will reveal obvious bias. However, this type of plot, with visual inspection, is insensitive to quantifying the bias between the method. A much more sensitive and useful indicator may be obtained by application of the *graphical linear* ratio method, previously described by Bretaudiere. This experiment should be performed at several control concentrations (as well as the normalized patient comparison) to distinguish systematic bias from proportional error.

### Une procédure pour évaluer la différence entre méthodes quantitatives analytiques

W. C. Griffiths *et al.*

Quand une nouvelle méthode clinique du laboratoire analytique est suffisamment précise et stable pendant une période de quelques semaines et qu'elle est linéaire et libre de toute contamination, quelques opérations simples permettent d'évaluer et de documenter son degré d'inexactitude. Le fait que la plupart des méthodes sont inexactes à un certain degré ne réduit pas leur valeur pour ceci. Cependant, c'est le laboratoire qui choisit une méthode qui doit connaître quelle est l'exactitude de cette méthode.

Une opération permet de déterminer la présence et la grandeur d'une différence entre la méthode étudiée et une seconde méthode dont les caractéristiques sont déja bien connues. Un grand nombre d'échantillons de malades sont analysés par les deux méthodes, de préférence le même jour. Une présentation graphique des résultats d'une méthode vers l'autre est généralement très informative et démontrera des différences entre les deux. Cependant, ce genre de présentation pour inspection visuelle se prête mal pour quantifier la différence entre les méthodes. Un indicateur plus sensible et utile peut être obtenu par l'application de la méthode 'graphical linear ratio' préalablement décrite par Bretaudière. Cette expérience devrait être effectuée pour différentes concentrations de contrôle (ainsi que les comparaisons normalisées pour malades) afin de différencier entre les différences systématiques et les erreurs proprotionnelles.

### Vorgehen für die Abschätzung systematischer Abweichungen quantitativier Analysenmethoden
W. C. Griffiths *et al.*

Falls eine neue klinische Analysenmethode genügend präzis und über eine Zeit von Wochen stabil ist, und linear und frei von Verschleppungsfehlern ist, dann können einige wenige einfache Operationen deren Grad der Ungenauigkeit erfassen und dokumentieren. Dass die meisten Methoden bis zu einem gewissen Grad ungenau sind, schliesst deren Verwendung nicht aus. Dagegen ist es Aufgabe des Labors, das die Methode auswählt, zu wissen, wie genau diese ist.

Eine der Operationen kann das Vorhandensein und das Ausmass der systematischen Abweichung zwischen einer vorliegenden und einer zweiten Methode, deren Eigenschaften als bekannt vorausgesetzt werden, bestimmen. Eine grosszügige Zahl von Patientenproben werden mit beiden Methoden analysiert, vorzugsweise jedes Paar am gleichen Tag. Eine grafische Darstellung der Resultate der einen Methode gegen die der anderen Methode ist normalerweise sehr informativ und wird eine offensichtliche systematische Abweichung aufzeigen. Jedoch erlaubt diese Methode mit visueller Beurteilung keine Qualifizierung der systematischen Abweichung der Methoden. Eine empfindlichere und nützlichere Indikation kann durch die Anwendung der Methode des grafischen linearen Verhältnisses erhalten werden, die bereits von Bretaudiere beschrieben wurde. Dieses Experiment sollte bei verschiedenen Kontrollkonzentrationen (als auch den normalisierten Patietenvergleichen) durchgeführt werden, um systematische Abweichungen von Proportionalfehlern zu unterscheiden.

### Page 151

### A concurrent process for the automatic preparation of biological samples combined with high-pressure liquid chromatographic analysis
D. C. Turnell and J. D. H. Cooper

A process for the pre-treatment of biological samples for high pressure liquid chromatographic analysis is described using automated sequential trace enrichment of dialysates. The design of the sample preparation unit is reported, together with the process algorithm required to integrate this device with the HPLC system. This enables the concurrent preparation of a sample during the chromatographic separation of the previously treated sample. This approach to sample preparation economically maximizes the use of HPLC equipment.

**La préparation automatique et simultanée d'échantillons biologiques et leur analyse par chromatographie liquide à haute pression**

D. C. Turnell and J. D. H. Cooper

Un processus pour la préparation d'échantillons biologiques pour l'analyse par chromatographie liquide à haute pression est décrit, utilisant l'enrichissement de traces séquenciel et automatique de solutions de dialyse. L'unité de préparation d'échantillons est présentée ensemble avec l'algorithme requis pour intégrer cette unité avec un système de HPLC. Ceci permet la préparation simultanée d'un échantillon pendant la séparation chromatographique de l'échantillon préparé au paravent. Ce système de préparation d'échantillons économique permet le meilleur usage de l'équipement HPLC.

**Gleichzeitige automatische Probenvorbereitung und Hochdruckflüssigchromatographie biologischer Proben**

D. C. Turnell and J. D. H. Cooper

Es wird über die Probenvorbereitung biologischer Proben dür die HPLC-Analyse unter Verwendung automatischer sequentieller Spurenanreicherung von Dialysaten berichtet. Die Konstruktion der Probenvorbereitungseinheit und die notwendigen Prozessalgorithmen für die Integration mit dem HPLC-System werden beschrieben. Das erlaubt die Probenvorbereitung gleichzeitig während der chromatographischen Trennung der vorgängig behandelten Proben. Dieses Vorgehen maximiert auf ökonomische Art die Auslastung von HPLC-Systemen.

---

*The following papers were published in Volume 8, Number 2 without the translations of the abstracts:*

**Page 56**

**Analyse par injection de flux (FIA): Un nouvel outil pour automatiser les processus d'extraction**

M. D. Luque de Castro

Un système d'extraction liquide-liquide basé sur l'analyse par injection de flux (FIA) est présenté. Ses propriétés principales, la théorie, les composantes de base, des applications et les avantages comparés à d'autres méthodes automatiques incorporant l'extraction en continue sont présentés.

**Flow Injection Analysis: Ein neues Werkzeug für die Automatisierung von Extraktionsprozessen**

M. D. Luque de Castro

Es wird eine Uebersicht zur flüssig-flüssig Extraktion mit FIA vorgestellt. Ebenso werden die wichtigsten Eigenschaften, Theorie, Grundeinheiten, Applikation und Vorteile gegenüber anderen Automationsmethoden (inklusive der kontinuierlichen Extraktion) angeführt.

**Page 49**

**Acquisition et traitement de données par ordinateur pour le dosage de la glucose, du lactate, pyruvate, alanine, glycérine et 3-hydroxybutyrate dans le sang humain par une méthode encymatique-fluorimétrique à flux continu**

C. S. Hetherington *et al.*

Un programme d'acquisition et de traitement de données utilisant un ordinateur Apple pour la détermination de la glucose, du lactate , pyruvate, alanine, glycérine et 3-hydroxybutyrate dans le sang humain est décrit. Les méthodes d'analyse enzymatique combinent des systèmes à flux continu avec la fluorimétrie. Le programme prévoit la possibilitié de correction pour la fluorescence présente dans les extraits de sang à l'acide perchlorique, mais peut, bien sûre, aussi être utilisé pour tous les dosages qui ne requièrent pas de mesures à blanc. Utilisant les mesures de la hauteur du pic pour déterminer la concentration des métabolites dans le sang, un logiciel consistant de 4 sous-programmes permet la flexibilité nécessaire, bien qu'une séquence rigide de groupes de standard, contrôle, échantillons soit nécessaire. Avec le sous-programme 'acquisition' les données de trois fluorimètres peuvent être acquises simultanément. Le sous-programme 'amend' est utilisé pour modifier les données par addition ou par élimination. Le sous-programme 'analyse' permet la détermination de la concentration de métabolites dans les extraits en acide perchlorique utilisant soit tous les standards soit des groupes de standard séparés. Le sous-programme collate' permet de collationner toutes les informations concernant les échantillons incluant le facteur de dilution, identification de malades et contrôle, et d'imprimer les résultats en un format cohérent. La corrélation entre les résultats déterminés par ordinateur et ceux mesurés par exploitation des traces de l'enregistreur est excellente. Le temps requis pour le traitement de données par l'ordinateur est nettement inférieur.

**Datenerfassung mit Rechner und Datenanalyse bei enzymatischen, fluorometrischen Durchflussmethoden für die Bestimmung von Glukose, Laktat, Pyruvat, Alanin, Glyzerol und 3-Hydroxybutyrat in menschlichem Blut**

C. S. Hetherington *et al.*

Es wird ein Programm beschrieben, das auf einem Apple Computer für die Bestimmung von Glukose, Laktat, Pyruvat, Alanin, Glyzerol und 3-Hydroxybutyrat in menschlichem Blut die Daten erfasst und verarbeitet. Die Analysenmethoden sind enzymatischer Art und kombinieren Fluorometrie mit kontinuierlichen Durchflusssystemen. Spezielle Aufmerksamkeit wird im Programm der Notwendigkeit geschenkt, für die Leerwerte der Fluoreszenz in Perchlorextrakten von Blut zu korrigieren, obwohl es auch für eine Reihe von Tests verwendet werden kann, wo Leerwertmessungen nicht erforderlich sind. Aus Peakhöhenmessungen wird die Blutmetabolitenkonzenentration in einem Softwarepaket, bestehend aus vier Teilen, errechnet. Dieses Paket erlaubt Flexibilität, obwohl eine starre Folge von sich wiederholenden Gruppen von Standardproben, Kontrollproben notwendig sind. Im Erfassungsteil können gleichzeitig Daten von drei Fluorometern erfasst werden. Im Vorverarbeitungsteil werden die Daten durch Hinzufügen oder Weglassen modifiziert. Im Analysenteil wird die Metabolitenkonzentration in den Perchlorsäure-Extrakten unter Verwendung der Mittelwerte aller oder verschiedener Standardgruppen bestimmt. Im Zusammenfassungsteil werden alle Probeninformationen inklusive Verdünnungsfaktoren, Patientenidentifikation und Kontrollen zusammengefasst und die Resultate in einem kohörenten Format ausgedruckt. Die Korrelation zwischen den Rechnerresultaten und solchen, die in konventioneller Art von Schreiberkurven abgelesen werden, ist ausgezeichnet. Der Zeitaufwand für die Verarbeitung der Daten mit dem Rechner ist wesentlich geringer.

### Page 70

**Automation d'un système à flux d'injection pour multiples échantillons**

J. Ruz *et al.*

Un système automatique 'flow injection' qui se base sur l'intégration de deux détecteurs (photométriques et potiontiométriques) est présenté. Ce système permet de collecter des données d'absorption et de pH de chaque échantillon moyennant un microprocesseur en ligne, qui, avec le programme de calcul nécessaire, procure la concentration de 9 espèces différentes de chrome présentes dans l'échantillon. Une configuration 'renversé' et un mode de mesure utilisant les zones asymétriques permet la discrimination entre les différents état d'oxydation du chrome.

**Automatisierung eines FIA-Systems für die Mulitkomponenten-analyse**
J. Ruz *et al.*

Ein automatisiertes Fliessinjektionsysstem, in welchem zwei Detektoren (photometrisch und potentiometrisch) integriert sind, erlaubt gleichzeitig mit einem Mikroprozessorsystem pH- und Absorptionsdaten zu erfassen. Mit einem geeigneten Rechenprogramm können damit die Konzentrationen von bis zu neun Arten von Chrom in der Probe bestimmt werden. Eine reversierte Konfiguration und das asymmetrische Mündungszonen-Vehalten werden für die Unterscheidung der verschiedenen Oxydationszustände des Chrom benützt.

---

### FORTHCOMING PAPERS

*Later issues of the Journal will contain the following articles:*

Time utilities: programs in MUMPS for calculating intervals of time and for timing events
*T. G. Pellar and A. R. Henderson*

A model for cost analysis—application to clinical laboratory test economics using computer facilities
*F. R. Hindriks, A. Bosman, Chr. Frowein, H. Kamps and W. van der Slik*

Biochemical laboratory management with a microcomputer
*R. Kowalezyk, G. Morgant, F. Ch. Baumann and J. Giboudeau*

Evaluation of a new semi-automated high-performance liquid chromatography method for glycolysated haemoglobins
*A. Carpinelli, A. Mosca and P. Bonini*

An evaluation of the Waters Pico-Tag system for the amino-acid analysis of food materials
*J. A. White, R. J. Hart and J. C. Fry*

Serum ionized calcium: evaluation of the analyte +2 calcium analyser in a clinical chemistry laboratory
*R. Freaney, O. M. Casey, T. Quinn and F. P. Muldowney*

Kinetic-based determinations in continuous-flow analysis
*M. D. Luque de Castro and M. Valcarcel*

Comparative evaluation of a Technicon SMAC2/RA1000 System with an American Monitor Parallel during normal service work
*A. J. Little et al.*

---

### NOTES FOR AUTHORS

Two copies of articles should be submitted to the Editor. All articles should be typed in double spacing with ample margins, on one side of the paper only. The following items should be sent: (1) a title-page including a brief and informative title, avoiding the word 'new' and its synonyms; a full list of authors with their affiliations and full addresses; (2) an abstract of about 250 words – this should succinctly describe the scope of the contribution and highlight significant findings or innovations; (3) the main text; (4) appendices (if any); (5) references; (6) tables, each table on a separate sheet and accompanied by a caption; (7) illustrations (diagrams, drawings and photographs) numbered in a single sequence from 1 upwards and with the author's name on the back of every illustration; captions to illustrations should be typed on a separate sheet.

**References**
References should be indicated in the text by numbers following the author's name, i.e. Skeggs [6].

**Illustrations**
Original copies of diagrams and drawings should be supplied, and should be drawn to be suitable for reduction to the page or column width of the *Journal*, i.e. to 85 mm or 179 mm, with special attention to lettering size.

**Proofs and offprints**
The principal or corresponding author will be sent galley proofs for checking and will receive 50 offprints free of charge.

*Manuscripts should be sent to either Dr P. B. Stockwell or Ms M. R. Stewart, see cover.*

